# Elevated Monocyte-to-High-Density Lipoprotein Ratio as an Indicator of Systemic Inflammation in Patients with Branch Retinal Vein Occlusion

**DOI:** 10.14744/bej.2021.94547

**Published:** 2021-09-24

**Authors:** Zeynep Duru, Orhan Altunel, Bedirhan Alabay, Ender Sirakaya, Ender Sirakaya, Bekir Kucuk, Musa Musaoglu

**Affiliations:** 1. Department of Ophthalmology, Kayseri City Hospital, Kayseri, Turkey; 2. Department of Ophthalmology, Kutahya Health Sciences University, Kutahya, Turkey; 3. Department of Ophthalmology, Yenikent State Hospital, Sakarya, Turkey

**Keywords:** Branch retinal vein occlusion, inflammation, monocyte-to-high-density lipoprotein ratio, MHR

## Abstract

**Objectives:**

This study was designed to assess the monocyte-to-high-density lipoprotein (HDL) ratio (MHR) as a possible marker of systemic inflammation in patients with branch retinal vein occlusion (BRVO).

**Methods:**

A study group of 62 patients with BRVO and a control group of 60 age-matched, healthy individuals were enrolled in the study. The blood lipid profile, hematology profile, and C-reactive protein (CRP) level were measured. The MHR was calculated as the ratio of the monocyte count to the HDL level, and the neutrophil-to-lymphocyte ratio (NLR) was calculated as the ratio of the neutrophil count to the lymphocyte count.

**Results:**

In patients with BRVO versus controls, the mean MHR was 14.1±5.1 vs 12.2±4.3 (p=.032), the mean NLR was 1.99±0.69 vs 2.01±0.86 (p=.889), and the mean CRP level was 3.44±2.53 mg/L vs 2.81±1.57 mg/L (p=.102). The area under the receiver operating characteristic curve for the MHR and the NLR was 0.621 and 0.519, respectively. The sensitivity and specificity of the MHR and the NLR to predict BRVO was 51% and 73% vs 79% and 35%, respectively.

**Conclusion:**

The MHR values were higher in patients with BRVO compared with those of the control group. BRVO seems to be associated with systemic inflammation.

## Introduction

The second most common vision-threatening vascular disorder in the retina, branch retinal vascular occlusion (BRVO) typically occurs at arteriovenous intersections where the venule and arteriole share an adventitial sheath (1). The increased rigidity of the crossing artery due to atherosclerotic disease may compress the underlying vein and cause turbulent blood flow, endothelial damage, and thrombus formation. Consequently, arterial hypertension and hypercholesterolemia are both known to contribute to atherogenesis and are defined as risk factors of BRVO (2-4).

Amid recently increased interest in how inflammation affects the pathogenesis of retinal vein occlusion, researchers have shown that local and systemic inflammation plays a role in the development of retinal vein occlusion by inducing both atherosclerosis and conditions for systemic hypercoagulability (5). Atherosclerosis is a low-grade, chronic inflammatory disorder characterized by a distinct pro-inflammatory TH1 cytokine pattern and the recruitment of T lymphocytes and monocytes to sites of inflammation (6). Lately, laboratory and clinical trials have also sought to elucidate the molecular pathways responsible for inflammation (7).

Due to the pro-inflammatory effects of monocytes and the anti-inflammatory effect of high-density lipoprotein (HDL), researchers have recently identified the ratio of monocytes to HDL (MHR) as a possible indicator of systemic inflammation. Previously, researchers have investigated the role of MHR in several well-known diseases (8-11). In this study, we aimed to evaluate MHR as a possible indicator of systemic inflammation in patients with BRVO.

## Methods

### Population and Design

Conducted in the department of ophthalmology, our cross-sectional study followed the tenets of the Declaration of Helsinki and received approval from the local ethics committee. All potential participants received both oral and written information about the study, and each willing participant provided his or her written informed consent to participate. The sample ultimately consisted of 62 patients with BRVO and, as controls, 60 healthy, age-matched individuals without any ocular or systemic disease, except for controlled hypertension, who had applied for pre-operative evaluations for the upper eyelid blepharoplasty surgery. Each participant underwent a comprehensive ophthalmic evaluation involving Goldmann applanation tonometry to measure intraocular pressure, Snellen charts to measure best-corrected visual acuity, slit-lamp biomicroscopy, and a dilated stereoscopic fundus examination. We diagnosed BRVO according to the results of stereoscopic fundus examinations for participants with retinal venous dilation and tortuosity accompanied by flame- and wedge-shaped regions of intraretinal hemorrhage.

### Exclusion Criteria

Ocular exclusion criteria for participation were any history of significant ocular disease, ocular surgery, uveitis, scleritis, retinal disease (except for BRVO), ocular trauma, or dense media opacities. Extraocular exclusion criteria were any history of systemic disease, including uncontrolled hypertension, renal failure, chronic obstructive pulmonary disease, hepatic disorders, anemia, malignancy, acute infectious disease, chronic systemic inflammatory disease, hyperlipidemia, thyroid abnormalities, cardiac disease, diabetes mellitus, autoimmune disease, and connective tissue disease. We also excluded all individuals who use tobacco, consume alcohol, or receive treatment involving any systemic medications that could affect hematological parameters (e.g., antihyperlipidemic therapy).

### Blood Samples

After performing the comprehensive ophthalmic examinations, we measured the blood lipid profiles, hematology profiles, and C-reactive protein (CRP) levels of participants. We collected all blood samples from the antecubital veins of participants between 09:00 and 11:00 a.m. after they had fasted overnight within 24 h of initial diagnosis of BRVO, and recorded all hematological parameters. We calculated MHR as the ratio of the monocyte count to the level of HDL and the neutrophil-to-lymphocyte ratio (NLR) as the ratio of the neutrophil count to the lymphocyte count.

### Statistical Analysis

We performed all statistical tests in the Statistical Package for the Social Sciences version 21 and present all data as mean±SD. For each continuous variable, we assessed normality using the Kolmogorov–Smirnov test. To compare results between the study and control groups, we subjected data of categorical variables to a Chi-square test and data of continuous variables to an independent t test. A receiver operating characteristic analysis was performed to determine the specificity and sensitivity of biomarker and the discriminative value of intergroup differences for MHR and NLR. Finally, we measured the Pearson correlation coefficient for correlation analysis and considered that all p<0.05 indicated statistical significance.

## Results

**Figure 1.**
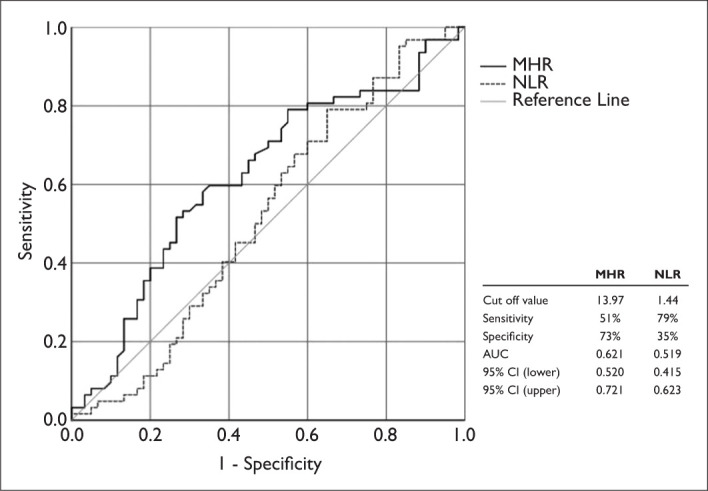
The receiver operating characteristics analysis for monocyte-to- high-density lipoprotein ratio (MHR) and neutrophil-to-lymphocyte ratio (NLR) in predicting branch retinal vein occlusion (BRVO). AUC: Area under the curve; CI: Confidence interval.

The sample consisted of 62 individuals with BRVO, aged 64.34±8.58 years (range: 47–85), and 60 healthy, age-matched individuals, aged 62.60±8.91 years (range: 48–81; p=.274). The baseline demographic characteristics (Table 1) and laboratory data (Table 2) between patients with BRVO and controls indicated no statistically significant difference between the groups. As shown in Table 3, mean MHR was 14.1±5.1 among patients with BRVO and 12.2±4.3 among controls (p=0.032), whereas mean NLR was 1.99±0.69 among patients with BRVO and 2.01±0.86 among controls (p=0.889). The results of correlation analysis (Table 4) revealed that MHR did not correlate with CRP or NLR in patients with BRVO. On the receiver operating characteristics curve, the area under the curve was 0.621 for MHR and 0.519 for NLR. The optimal cutoff value was 13.97 with 51% sensitivity and 73% specificity for MHR and 1.44 with 79% sensitivity and 35% specificity for NLR (Fig. 1).

**Table 1.** Baseline characteristics of patients with branch retinal vein occlusion (BRVO, n=62) and healthy, age-matched controls (n=60)

Parameters	BRVO	Control	p
Sex, n (%)			
Female	28 (45)	31 (52)	.472*
Male	34 (55)	29 (48)	
Hypertension, n (%)			
Positive	44 (71)	38 (63)	.369^*^
Negative	18 (29)	22 (37)	
Age, year			
Mean±SD	64.34±8.58	62.60±8.91	.274**
Range	47-85	48-81	

SD: Standard deviation; *: Chi-square test, **: Independent t-test.

**Table 2.** Laboratory data of patients with branch retinal vein occlusion (BRVO, n=62) and healthy, age-matched controls (n=60)

**Variables**	**BRVO**	**Control**	p*
	Mean±SD	Mean±SD	
Neutrophil count, 10^3^/lL	4.45±1.05	4.14±1.03	.103
Lymphocyte count, 10^3^/lL	2.36±0.53	2.24±0.59	.272
Monocyte count, 10^3^/lL	0.60±0.16	0.55±0.14	.118
Platelet count, 10^3^/lL	257.5±49.9	255.8±41.7	.836
Haemoglobin (g/dL)	14.28±1.72	14.63±1.99	.294
Hematocrit (%)	42.61±4.23	43.98±5.06	.108
Total cholesterol (mg/dL)	193.29±35.66	196.80±45.16	.634
HDL cholesterol (mg/dL)	45.15±10.18	47.63±8.38	.144
LDL cholesterol (mg/dL)	115.69±33.46	118.68±35.76	.634
Triglycerides (mg/dL)	162.23±62.37	152.43±65.38	.399
C-reactive protein (mg/dL)	3.44±2.53	2.81±1.57	.102
ESR, (mm/h)	9.61±4.53	9.03±4.47	.479

HDL: High-density lipoprotein; LDL: Low-density lipoprotein; ESR: Erythrocyte sedimentation rate; SD: Standard deviation; *: Independent t-test.

**Table 3.** Monocyte-to-high density lipoprotein ratio (MHR) and neutrophil-to-lymphocyte ratio (NLR) between patients with branch retinal vein occlusion (BRVO, n=62) and healthy, age-matched controls (n=60)

**Variables**	**BRVO**	**Control**	p*
	Mean±SD	Mean±SD	
MHR	14.1±5.1	12.2±4.3	.032
NLR	1.99±0.69	2.01±0.86	.889

*: Independent t-test. SD: Standard deviation.

**Table 4.** Correlation analysis between MHR and other laboratory data among patients with branch retinal vein occlusion (BRVO, n=62)

	**MHR**	
	**R**	p*
Neutrophil	.195	.129
Lymphocyte	.166	.197
Platelet	-.169	.188
Haemoglobin	.218	.088
Hematocrit	.200	.119
Total cholesterol	-.055	.669
LDL cholesterol	-.038	.767
Triglycerides	.121	.347
C-reactive protein	.032	.805
ESR	-.243	.057
NLR	-.007	.957

NLR: Neutrophil/lymphocyte ratio; LDL: Low-density lipoprotein; ESR: Erythrocyte sedimentation rate. *: Pearson correlation test.

## Discussion

Local and systemic types of inflammation are important etiological factors of BRVO, among which vascular and inflammatory mediators are especially salient, and systemic inflammation is thought to play a particularly important role in the condition’s etiology (12-14). Predisposing systemic risk factors for BRVO include dyslipidemia, high levels of plasma homocysteine, hypertension, and diabetes, all of which are also independently associated with atherosclerosis, a disease whose relationship with BRVO has been studied in detail (2-5). Initial pathological findings of atherosclerosis indicate T-lymphocytes and monocyte-derived macrophages that later progress to form thrombi and clots (15).

In recent studies, researchers have investigated MHR in relation to diseases such as chronic kidney disease, cardiovascular disease, and endothelial dysfunction (16-20). In inflammatory conditions, monocytes play an important role in the release of pro-inflammatory and pro-oxidative cytokines. By contrast, HDL cholesterol molecules inhibit the migration of monocytes in response to oxidized low-density lipoprotein and the expression of endothelial adhesion proteins, and consequently, HDL exhibits anti-inflammatory properties (21). Consequently, MHR has emerged as a potential indicator of inflammation calculated by dividing monocyte counts amid inflammation by the level of anti-inflammatory HDL. Therefore, our study aimed to clarify the association between MHR and BRVO.

We evaluated MHR as a different marker of systemic inflammation in patients with BRVO. Among our results, MHR was significantly higher among patients with BRVO than controls, although the between-group difference in NLR was not statistically significant. Patients with BRVO also exhibited CRP levels higher than those among controls, although not to a statistically significant degree. In the previous studies conducted to evaluate laboratory findings representing patients with BRVO, Kumral et al. (22) assessed mean platelet volume (MPV) and NLR; since neither of them seemed to be affected by BRVO, their results suggested that NLR cannot be used to predict inflammation in relation to BRVO. Conversely, Dursun et al. (23) reported that higher NLR is associated with the development of retinal vein occlusion, the risk of which NLR might be used to predict. In another study, Onder et al. (24) evaluated MPV in BRVO and reported that MPV was significantly higher in patients with hypertensive BRVO, which contradicted the results of Kumral et al. Recently, Şatırtav et al. (25) reported that elevated MHR is significantly associated with BRVO, which was consistent with our study.

Given those findings and our results, we suggest that MHR is a better indicator of systemic inflammation than other hematological parameters in inflammatory disorders, largely because MHR indicates the balance of pro-inflammatory and anti-inflammatory reactions. Monocytes and macrophages, both responsible for pro-inflammatory and pro-oxidant reactions, play a key role in inflammatory reactions because their activation prompts inflammatory cytokine synthesis. Conversely, HDL decreases macrophage accumulation, inhibits the migration of monocytes, increases the expression of nitric oxide synthase in endothelial tissues, protects endothelial cells, and exhibits antioxidant and anti-inflammatory activity (26). Consequently, MHR might be more predictive of systemic inflammation than other markers.

Our study had also one limitation. We used a simple baseline determination that might not reflect a patient’s long-term status.

Altogether, inflammation seems to play an important role in BRVO. We suggest that MHR can be a useful, practical, inexpensive, and easily measured indicator of systemic inflammation to predict the risk of developing BRVO. However, further studies are necessary to evaluate the role of MHR on prognosis and treatment response of BRVO in larger samples.

## Disclosures

### Ethics Committee Approval:

Erciyes University Ethics Committee, 2019/296, 08.06.2019.

### Peer-review:

Externally peer-reviewed.

### Conflict of Interest:

None declared.

### Authorship Contributions:

Involved in design and conduct of the study (ZD, OA); preparation and review of the study (ZD, OA); data collection (BA, ES); and statistical analysis (BK, MM).
